# Artificial intelligence and Italian culture: an understanding of how artificial intelligence can transform the radiation therapy landscape

**DOI:** 10.1007/s12032-023-02154-y

**Published:** 2023-09-04

**Authors:** Francesca De Felice

**Affiliations:** Department of Radiotherapy, Department of Radiological, Oncological and Pathological Sciences, Radiation Oncology, Policlinico Umberto I, “Sapienza” University of Rome, Viale Regina Elena 326, 00161 Rome, Italy

**Keywords:** Artificial intelligence, Machine learning, Radiation therapy, Cancer, Treatment, Model, Omica

## Abstract

The aim is to support the perception of artificial intelligence in the radiation therapy landscape.

Artificial intelligence (AI) is becoming part of medical daily clinical practice and is expected to be ever more integrated in the next years to come. Clinicians and researchers are interested how AI systems can improve their own sector. It is important to support their perception of AI, favouring a real understanding of how AI is used and how it works. Probably their AI technical understanding is patchy and should be educated by the imaginary narratives that are popular in their mind. It represents a crucial step to develop new technologies and orient research projects [1].

In this context, we take a unique approach to exploring AI's potential in the radiotherapy field using the charm of Italian culture. The hope is to cultivate a deeper understanding of AI and its role in strategic decision-making and innovative applications. For this purposes we concentrate on two popular figurative associations.

The first one is linked to the pizza and its process from *bench to bedside* we can say [2]. Being an esteemed *pizza maker* implies to use optimal ingredients and be extremely precise in time cooking. Before deliver the pizza around and share own recipes, people (friends first and enemies after!) have to eat and approve it. And so each step can be related to AI process [3]: (i) ingredient preparation and gourmet ingredients refer to features curation and selection; (ii) time-cooking refers to model building; (iii) friends and enemies who eat pizza represent evaluation in a test cohort and external validation, respectively; (iv) deliver pizza around refers to reproducibility of the model; (v) share recipes implies an open source code of the model, better if user-friendly. Therefore, an AI (radiation) oncologist must be firstly accurate in data collection and then in processing it.

The second figurative association is linked to the poet Dante Alighieri and his view of the *fortune tellers*, who travelled by walking backwards because they could see only backwards, not forwards [4, 5]. Similarly, when we use AI, we look at retrospective data hopefully in a dynamic collection. Some will argue that static models are easier to maintain than dynamic models, but if we consider that oncologic scenario evolves at a much faster pace than a typical clinical trial, this makes it even more important to have accurate up-to-date model [6]. Therefore, a static model tend to be outdated and not aligned with reality and this would be unacceptable. The dynamic nature of the process should be emphasized. The information collected onto an AI-based model should be implemented continuously to resolve the real problems in patient management. It should be necessary to zoom out to see well-known baseline characteristics—such as TNM stage, risk factors, performance status—and zoom in to add more details—such as “*omics*” features and biomarkers–. This implies that data collection cannot be static. The perceived complexity is partly inevitable. Nevertheless, an interdisciplinary group, including clinicians and computer scientists, can help to improve the process models. To guarantee a clear interpretation of the model, the input of a dataset should not be arbitrary as the output should have the same meaning across different models. It is important to use conventions in a consistent manner.

To conclude, we suggest adopting ideas from *pizza maker* and *fortune tellers*, in order to use AI in a responsible manner. To turn predictions into recommendations, process mining should be the next step to force clinicians to work with a specific method and guarantee a level of accuracy of the model.
